# Structured Observations Reveal Slow HIV-1 CTL Escape

**DOI:** 10.1371/journal.pgen.1004914

**Published:** 2015-02-02

**Authors:** Hannah E. Roberts, Jacob Hurst, Nicola Robinson, Helen Brown, Peter Flanagan, Laura Vass, Sarah Fidler, Jonathan Weber, Abdel Babiker, Rodney E. Phillips, Angela R. McLean, John Frater, SPARTAC trial investigators

**Affiliations:** 1 The Peter Medawar Building for Pathogen Research, Nuffield Department of Clinical Medicine, Oxford University, Oxford, United Kingdom; 2 The Institute for Emerging Infections, The Oxford Martin School, Oxford, Oxford United Kingdom; 3 Oxford NIHR Comprehensive Biomedical Research Centre, Oxford, United Kingdom; 4 Division of Medicine, Wright Fleming Institute, Imperial College, London, United Kingdom; 5 Medical Research Council Clinical Trials Unit, London, United Kingdom; 6 Department of Zoology, Oxford University, Oxford, United Kingdom; National Institute of Genetics, JAPAN

## Abstract

The existence of viral variants that escape from the selection pressures imposed by cytotoxic T-lymphocytes (CTLs) in HIV-1 infection is well documented, but it is unclear when they arise, with reported measures of the time to escape in individuals ranging from days to years. A study of participants enrolled in the SPARTAC (Short Pulse Anti-Retroviral Therapy at HIV Seroconversion) clinical trial allowed direct observation of the evolution of CTL escape variants in 125 adults with primary HIV-1 infection observed for up to three years. Patient HLA-type, longitudinal CD8+ T-cell responses measured by IFN-γ ELISpot and longitudinal HIV-1 *gag*, *pol*, and *nef* sequence data were used to study the timing and prevalence of CTL escape in the participants whilst untreated. Results showed that sequence variation within CTL epitopes at the first time point (within six months of the estimated date of seroconversion) was consistent with most mutations being transmitted in the infecting viral strain rather than with escape arising within the first few weeks of infection. Escape arose throughout the first three years of infection, but slowly and steadily. Approximately one third of patients did not drive any new escape in an HLA-restricted epitope in just under two years. Patients driving several escape mutations during these two years were rare and the median and modal numbers of new escape events in each patient were one and zero respectively. Survival analysis of time to escape found that possession of a protective HLA type significantly reduced time to first escape in a patient (p = 0.01), and epitopes escaped faster in the face of a measurable CD8+ ELISpot response (p = 0.001). However, even in an HLA matched host who mounted a measurable, specific, CD8+ response the average time before the targeted epitope evolved an escape mutation was longer than two years.

## Author Summary

The cytotoxic T-lymphocyte (CTL) arm of the immune response is thought to play a significant role in the control of HIV-1 infection. Mutations within the HIV-1 genome allow the virus to escape recognition by CTLs and so evade the immune response. These escape mutations have been well documented but observed waiting times to escape within an individual have ranged from days to years. Many studies describing CTL escape have taken a detailed look at a few patients. Our analysis is based on a cohort of 125 clinical trial participants with immunologic and viral sequence data taken at regular longitudinal time points within the first few years of infection. Results suggested that the majority of CTL-related mutations present early in infection had been transmitted in the infecting viral strain as opposed to arising in the new host due to selection pressure imposed by CTLs. Whilst the prevalence of CTL escape mutations in the dataset was high, the incidence of new escape was relatively low; around one third of patients did not drive an escape within the first two years. Patients possessing a ‘protective’ HLA genotype had a significantly shorter waiting time to first escape than those without.

## Introduction

The HIV-1-specific cytotoxic T-lymphocyte (CTL) response begins as early as 2 to 3 weeks after infection [1], and there is evidence to suggest it may play an important role in the early control of viraemia [2, 3]. The onset of the response coincides with the decline of viral load. Certain host HLA genotypes (and therefore certain specific responses) have been found to be significantly associated with delayed progression to AIDS [4–6]. In addition to this, some studies based on the depletion of CD8^+^ T-cells in SIV-infected macaques have shown that the CTL response makes a crucial contribution to viral control in this animal model of AIDS [7]. However, others have questioned the role of CTL in controlling productive infection, both in the SIV-macaque model and in humans [8, 9].

Many studies have demonstrated the capability of the HIV-1-specific CTL response to select for viral variants that escape recognition by CD8^+^ T-cells or prevent antigen presentation by HLA class I molecules and so evade the immune response [10–14]. Indeed, work done to quantify the effects of the various selective forces acting on HIV has found that 53% of non-*env* mutations that rise to fixation in the first few years represent viral adaptation to CD8^+^ T-cell responses [15] whilst a detailed deep sequencing study of one patient found that the majority of early mutations were CTL related [16].

CTL escape has been observed to occur throughout the different stages of HIV-1 infection, from soon after seroconversion [13, 17–20] to many years into chronic infection [11, 21, 22]. Some epitopes consistently escape earlier and more frequently than others [23–26] but time to escape also varies considerably between patients and is likely to be influenced by factors such as strength and quality of the CD8^+^ T-cell response as well as pre-existence of compensatory mutations in sequences surrounding epitopes [11, 21, 27]. Escape pathways are complex; in many cases low frequency variants arise early, replacing the transmitted sequence, but it is not until some time later that one escape mutant, often containing multiple amino acid mutations, emerges and rises to fixation [2, 28–30]. In the absence of selective pressure from CTLs, reversion of escape mutations back to wild type has been documented [20, 31].

In this study we analysed the timing and extent of CTL escape in 125 patients across the HIV-1 *gag*, *pol*, *nef* and *env* genes. We used longitudinal viral sequence data covering the first three years of infection to count escape events in each of 46 epitopes in all available patient samples, and only considered periods when patients were off therapy. Approximately one third of patients drove escape in an epitope for which they were HLA-matched within their first year of infection and escape continued to be seen into the second year. However an estimated 33% of patients had still not driven any escape in their HLA-restricted epitopes across *gag*, *pol* or *nef* by the end of their second year off therapy. In looking at whether escape was clustered in patients, we found little evidence to suggest that this was the case. We also observed that time to escape for individual epitopes was faster in HLA-matched patients and in patients who were able to mount a measurable CD8^+^ T-cell response, and that patients with protective HLA alleles were more likely to have their first escape sooner.

## Results

### Cohort characteristics

The cohort consisted of 125 treatment-naive, HIV-1 subtype B infected adults who were recruited soon after the estimated date of seroconversion (median 11 weeks; range 1–20 weeks) as part of the SPARTAC clinical trial and followed for up to three years (see Methods, full details in [32], patient characteristics shown in Table 1). Participants were randomised into one of three arms, which determined whether they received no treatment, 12 weeks antiretroviral therapy (ART) or 48 weeks ART. Consensus viral sequences for four immunogenic genes (*gag*, *pol*, *env* and *nef*), and IFN-γ CD8^+^ T-cell ELISpot data were collected longitudinally. Participants were followed for up to three years whilst off ART (median 109 weeks, IQR 85–145 weeks); some participants initiated long-term ART before the end of the three years, upon reaching the trial clinical endpoint. The amount of data available at each time point is summarised in S1 Table.

**Table 1:** Patient characteristics table.

**Characteristic**	**Whole trial (N = 366)**	**UK patients (N = 125)**
**Sex—no. (%)**		
Male	219 (60)	125 (100)
Female	147 (40)	0 (0)
**Age—yr**		
Median (interquartile range)	32 (25–40)	34 (29–42)
Range	19–63	19–60
**Estimated time since seroconversion at randomisation—wk**		
Median (interquartile range)	12 (9–15)	11 (7–14)
Range	1–24	1–20
**CD4+ count—** cells/mm^3^		
Median (interquartile range)	559 (435–700)	550 (435–682)
Range	95–1399	130–1310
**HIV RNA level—copies/ml**		
Median (interquartile range)	4.53 (3.67–5.18)	4.78 (4.08–5.29)
Range	1.40–6.47	2.22–6.22

For this study, we took all the UK HIV-1 Subtype B infected participants that had been enrolled in the SPARTAC trial and for which exact times of initiation and cessation of both short course ART and, if applicable, long term ART were known. The resulting cohort of 125 participants had similar characteristics to the whole trial cohort except that all the UK HIV-1B infected participants recruited were male. Participants in the UK cohort were also recruited slightly earlier in infection on average, and (perhaps consequently) had slightly higher viral loads on average at recruitment compared to the trial as a whole.

### Escape prevalence at baseline and after 1 year

A CTL escape mutation was defined as *any amino acid variation in previously identified (and phenotypically confirmed) escape sites within optimal defined epitopes* in *gag*, *pol*, *env* or *nef* (see Methods for more details). All analyses were also run using alternative, more lenient definitions of escape. Those additional analyses, which are shown in S7–S12 Figs., confirm that the results presented here are not dependent on this definition of escape. In addition to drawing on a large body of collated literature to form this definition we also validated it by analysing the strength of patients’ CD8^+^ T-cell responses to autologous epitope variants (see Methods and S2 Table).

**Figure 1:**
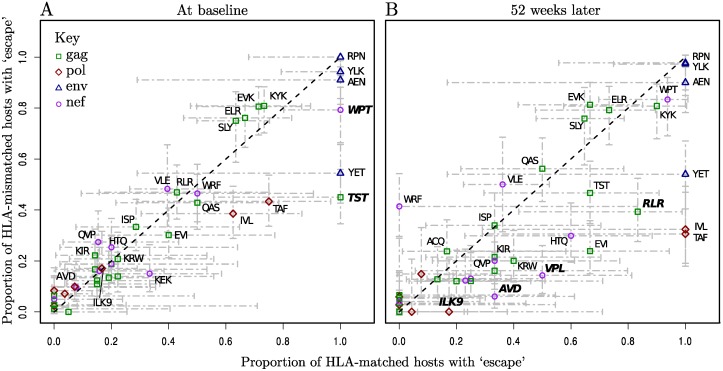
Escape prevalence in HLA-matched and-mismatched hosts. Epitopes are labelled where possible with their first three amino acids, and length if necessary to distinguish them. Bars represent 95% binomial confidence limits (Agresti-Coull method). The dotted black line is *y* = *x*. Epitopes labelled in bold have a significantly higher proportion of escape in matched hosts than in mismatched hosts (one-tailed Fisher’s Exact test, p < 0.1, note that multiple testing was not corrected for since the purpose of these tests was simply to give a relative measure of significance). Raw data for the number of patients in each category can be found in S3 and S4 Tables. First visit (baseline) sequence data was available in at least one gene for 122/125 patients. The range of time from seroconversion among these patients was 1–20 weeks, median 11. (A) At baseline the majority of epitopes have escape at equal prevalence in HLA-matched and-mismatched hosts (B) 52 weeks later within-host evolution has resulted in a higher prevalence of escape in HLA-matched hosts for many epitopes, and in the distribution as a whole HLA-matching and escape are now significantly associated (p < 0.01, permutation test, see Methods for details).

At baseline (the first trial study visit) the data comprised a total of 3831 epitope sequences from 122 patients in which we were able to look for variation within known escape sites. The range of times from seroconversion amongst these patients matched that of the whole cohort. Of these, 1235 contained escape mutations. However, this escape at baseline was, for almost all epitopes, equally prevalent in HLA-matched and HLA-mismatched hosts (Fig. 1A). This pattern is more consistent with variation that has been transmitted than variation that has been driven by within-host HLA-restricted immunity prior to the first sampling. In contrast, after one year, four epitopes derived from *gag*, *pol* and *nef* had markedly higher escape prevalence in HLA-matched hosts than in mismatched hosts (Fig. 1B). Further analysis (see Methods for details) confirmed that the distribution of points in Fig. 1B (at one year) is significantly different from what would be expected under the null hypothesis that escape is randomly distributed between matched and mismatched hosts, where as the distribution at baseline is not.

### Incidence of escape during up to three years of observation

**Figure 2:**
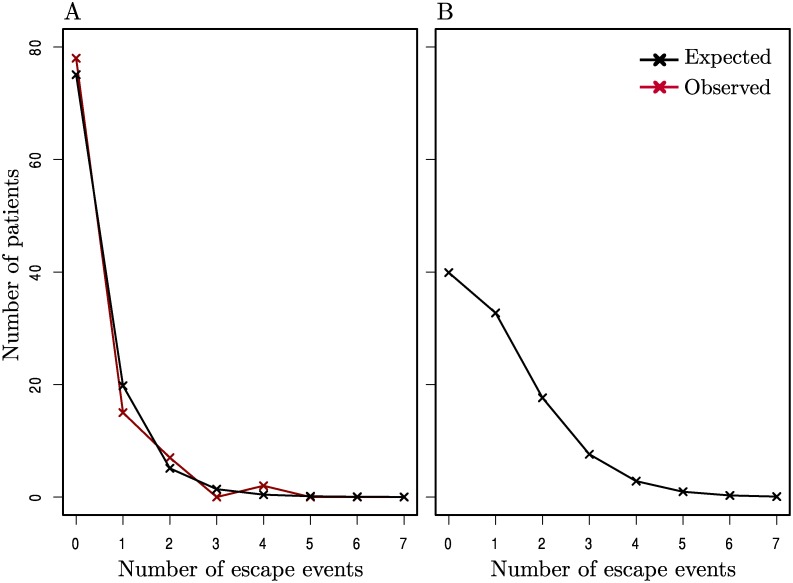
The distribution of incident escape across patients. (A) The observed distribution of incident escape (in HLA-matched patient-epitope pairs) across patients (red line) is similar to the distribution given by a Poisson process where the average rate of escape is constant across all patient-epitope pairs (black line). (B) The expected distribution of escape supposing all patients in (A) had a full set of sequence data and were observed for a full 2 years whilst off-ART. A goodness-of-fit test showed that there was no significant difference between the observed and expected distributions in (A) (p > 0.1). Details of calculations for the expected distributions are given in the Methods.

Of those epitopes sequenced, 2297 were initially wild-type and had at least one later sequence for comparison, and hence were candidates to allow the observation of incident escape (note that in a few cases this initial time point was after baseline due to sequence data being unavailable). Due to the high variability of the *env* gene, the vast majority of epitopes in *env* (all 32 HLA-matched patient-epitope pairs and 215/316 HLA-mismatched patient-epitope pairs) were already escaped at baseline, and hence whilst these were included in our analyses we were not able to comprehensively study incident escape in *env*. In the 2297 candidate epitopes, just 108 ‘escape’ events were observed in patients studied while off ART and only 37 of those were in epitopes in an HLA-matched patient. To see if there was evidence for a separate group of particularly fast escaping patients we compared the distribution of the number of escape events per host (in their HLA-restricted epitopes) with a random distribution, calculated using a Poisson process, and found no significant difference between the distributions (Fig. 2A). In fact this Poisson process in which escape was driven at an average rate of 0.0018 per week across all HLA-matched patient-epitope pairs, described the distribution of incident escape across patients well. Since many patients had missing data, particularly at later time points (S1 Table) we used this average rate to construct a similar expected distribution supposing all patients had had a full set of sequence data for the first two years (whilst untreated) (Fig. 2B). Even after accounting for the missing data in this way, only 12% of patients would have driven more than two escape variants in HLA-restricted epitopes and the median and modal numbers of incident escape events were one and zero respectively.

This analysis is meaningful if patients with missing data would have had similar rates of escape to those observed for a full two years whilst untreated. We believe this to be a valid assumption and assess it in further detail later.

### When does escape happen?

Time to escape can be described either for each patient in a population (how long does it take until a viral variant with a new escape mutation is detectable in a patient?) or for each epitope within each patient (how long does it take until an escape mutation appears in an initially wild-type epitope?). By considering the first such escape event (in patients or in epitopes) the well-developed statistical tools of survival analysis become applicable. Since only 9% of patients have more than one incident escape event in an epitope for which they are HLA-matched (Fig. 2A) very little information is lost in such analysis.

In assessing the suitability of these analyses we also needed to address the question of whether the time at which patients are censored from the survival analysis (which occurs when they have no further sequence data) is independent of the rate at which they drive escape mutations. For some patients the fact that we were unable to obtain sequence data at later time points was a direct consequence of the fact that they had reached the trial primary endpoint (a CD4 cell count < 350 cells /μ l or commencing long-term ART) within the time frame of our observation period. Hence if, as some might hypothesise, escape events can drive clinical failure, it is possible that censored patients would have high rates of escape that we’d fail to observe.

We looked in more detail at those patients who reached the trial endpoint (or clinically ‘failed’) within our three-year observation period. An incident escape was observed in 6/20 patients for whom we had no sequence data after clinical failure compared to 7/24 patients who did have sequence data after clinical failure. In the 7 patients in the latter group who drove at least one escape event, there were just two cases where an escape mutation was first seen at the time point after clinical failure (see S1 Text and S2 Fig. for more details). Therefore the extent to which escape is linked to censoring time is small, and it is fair to assume that the (unobserved) rate of escape in patients who are censored from the analysis is the same as that of those who continue to be observed.

**Figure 3:**
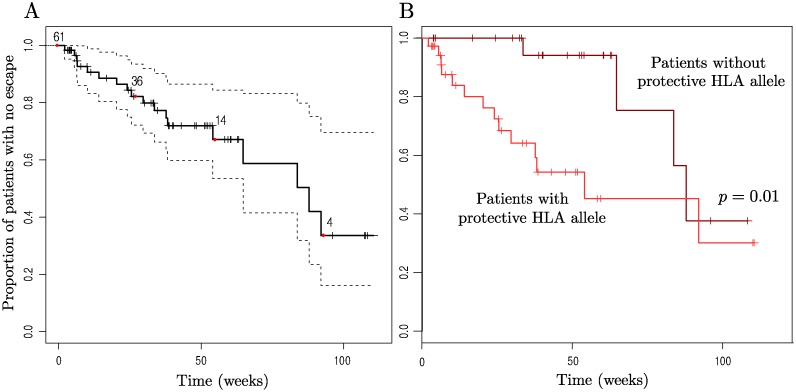
Kaplan-Meier plots of time to first escape using a midpoint approximation. Patients who were either missing data for *gag* or *nef*, the two genes for which the most data was available, were not included so as not to skew the results due to lack of data. 4 of these patients had no epitopes restricted by their HLA types that were WT at baseline, so *n* = 61 initially here. (A) Time to first escape in an HLA-restricted epitope (solid line) is plotted along with the 95% confidence intervals (dotted lines). (B) Patients are split according to whether they have one of the more ‘protective’ HLA alleles or not. The set of beneficial alleles was taken to be B*58, B*27, B*57, A*26, B*51, A*11, B*14, B*18, B*08 (all the HLA-A and-B alleles down to B*08 that are present in our data, taken from the ranking in [47]) as this split the patients approximately in half. Having a protective HLA resulted in a significantly increased risk of HLA-matched escape (p = 0.01, Likelihood Ratio test on Cox Proportional Hazards model with single predictor. Hazard ratio = 3.7, 95% C.I. = (1.2, 11.3)). In both plots the x-axis represents the time since cessation of treatment, or baseline for those not receiving treatment, and vertical checks mark time points at which patients were censored (either because they began long term ART or because there was no further sequence data available for them). Numbers indicate the number of patients who have not yet escaped or been censored at the corresponding time points marked by red dots (0, 27, 55, 93 wks).

Fig. 3 shows time to first escape for patients whilst they were off ART. Only patients with at least two time points of sequence data for both *nef* and *gag* (the genes for which the most data were available) were used to avoid underestimating the occurrence of escape due to missing data. This left 65 patients for this analysis. Note that this number includes both treated and untreated patients (range of previous ART duration 2–12 months). We also considered these patient groups separately (S4–S6 Figs.) and this did not change our conclusions. Multivariate Cox models for time to escape confirmed that the impact of early limited treatment on CTL escape was negligible (S1 Text).

The Kaplan-Meier plot for time to first escape in an HLA-restricted epitope shows that there is large variation between patients in time to first escape, with some patients who received: 12 weeks of therapy escaping before the 24 week time point (so that the midpoint approximation gives the therapy-adjusted time to escape as around 6 weeks, assuming no viral evolution on ART) whilst an estimated 33% of patients did not drive any new escape in their HLA-restricted epitopes in 92 weeks off ART (95% C.I. (16,70)%). Note also that the slope of the curve is close to constant; there is no indication of a propensity for many incident escape events to arise early during the observation period and fewer later.

### Having a protective HLA type is associated with earlier first escape

Although there was no evidence for a separate group of patients with a high frequency of escape (Fig. 2), there was evidence that some patients had a tendency to drive escape mutations sooner than others. Fig. 3B shows a Kaplan-Meier plot for time to first HLA-matched escape event for patients split according to whether or not they have one of the more protective HLA types. First escape is earlier in patients with a ‘protective’ HLA allele than in those without.

### Waiting time to escape for a wild-type epitope

**Figure 4:**
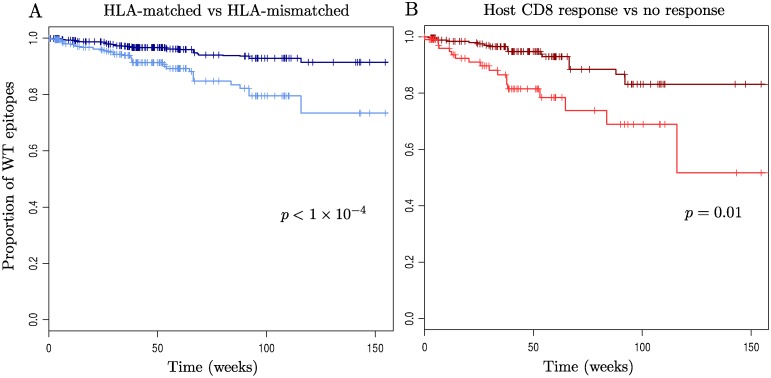
Kaplan-Meier survival curves for wild-type epitopes. For the 114 patients and 46 epitopes for which appropriate sequence data was available: (A) Time to escape in epitopes within HLA-mismatched (dark blue) and-matched (light blue) hosts is shown. (B) Epitopes in HLA-matched hosts are split according to whether they have a measurable CD8^+^ T-cell ELISpot response to them (light red) or not (dark red). Escape is strongly significantly faster in epitopes within HLA-matched hosts (p < 1×10^-4^), hazard ratio = 2.6, 95% C.I. = (1.7, 4.2)) and then faster again if the host has a measurable CD8^+^ T-cell ELISpot response to that epitope (p = 0.013, hazard ratio = 3.0, 95% C.I = (1.3, 7.0)). Note that p-values and hazard ratios were calculated using a mixed effects model with random intercept terms for both patient and epitope ID, to account for correlation of observations within patients and within epitopes (see Methods).

Fig. 4 records Kaplan-Meier survival curves for wild-type epitopes. This analysis included all patient epitope pairs which were observed whilst the patient was off ART (i.e. in which it would have been possible to observe escape), giving a total of 46 epitopes and 114 patients. Here ‘failure’ is the appearance of an escape mutation in an epitope. The characteristic time to escape for a wild-type epitope is of the order of months or years. It is faster in HLA-matched hosts (Fig. 4A), and amongst epitopes in HLA-matched hosts it is faster if there is a detectable CD8^+^ T-cell ELISpot response to that epitope in that host (Fig. 4B). Yet even in a host with the epitope’s restricting HLA and a detectable CD8^+^ T-cell response more than half of wild-type epitopes still show no escape after two years’ observation (Fig. 4B, survival = 0.69 between 84 and 115 weeks).

## Discussion

The literature on CTL escape in HIV-1 infection is dominated by studies of small numbers of individuals [17, 18, 33–35]. Several publications describe single patients in whom many CTL escape mutations arise and recent papers have described very active, rapid outgrowth of many escape mutations in a small number of patients studied intensively in the first weeks of infection [2, 19]. It has been known for some time that escape mutations that arise in early infection have a faster fixation rate than those that arise during chronic infection [10, 36]. These observations lead to a natural set of questions about the timing of CTL escape in HIV-1 infection. What is a normal number of escape events observable in a patient during the first few years of infection? Is escape very concentrated amongst a subset of patients so that patients fall into two groups—one with lots of escape and one with little? Are escape events also focussed in time so that much escape occurs early in infection and very little later on?

To address questions like this properly it is necessary to follow a large number of patients through time, so that those in whom nothing happens are given proper attention. This study of 125 patients in the first few years of untreated infection from a large randomised controlled trial does just that. It finds that the incidence of escape is very low; after two years of observation an estimated 33% of patients had not seen a first escape incident in *gag*, *pol* or *nef*. The observed frequency of incident escape across all epitopes in HLA-matched hosts translated to an estimated 12% of patients driving more than 2 escape mutations within 2 years (Fig. 2B). The inevitable conclusion is that patients with many escape events are unusual and, further, there was no evidence for a separate group of patients with a very high number of escape events (Fig. 2A). Equally, the distribution of escape through time showed no evidence for much escape early on and little later; the Kaplan Meier survival curves for the proportion of patients without an escape, and the proportion of epitopes without escape both have constant slopes (Figs. 3 & 4).

It has long been known that some HLA types confer a survival advantage upon their host. The relationship between the restricting HLA type and the propensity for an epitope to escape has been contentious. We previously [37] analysed the prevalence of escape in 84 chronically infected patients and concluded that protective T-cell responses were associated with more frequent viral escape, hypothesising that this result was due to a high fitness cost of mutations in epitopes restricted by protective alleles. However, Asquith [38], in a study that involved measuring the entropy of epitopes across sequences in the LANL HIV database, found that epitope entropy was significantly positively correlated with relative hazard of the restricting HLA allele, concluding that protective HLA alleles restricted epitopes that escape less frequently than average. In this cohort patients with protective HLA alleles escape significantly sooner (Fig. 3B), and epitopes restricted by protective HLAs escape faster (although not significantly so) than others (S3 Fig.). Further, all patients with escape in two or more HLA restricted epitopes had at least one of our defined list of protective HLAs. This large cohort study thus supports our previous finding that protective HLAs are associated with more frequent escape.

At first glance it seems counterintuitive that patients with some HLA types escape sooner and more frequently than others but that there was no evidence for a separate group of patients with much escape. However this apparent conflict is easily resolved; it is likely that patients with protective HLAs drive escape more frequently but do not have so much escape as to form a distinct subset of patients. They just lie at the top end of the distribution.

Why is escape in this cohort so slow? Is it because we missed very early escape events that occurred before patients were recruited? Is it that escape is concentrated in a small number of epitopes in which the process of escape is already largely complete? Or is it that escape is rare because responses are rare? Finally, is the definition of escape used here too restrictive?

Liu et al [19] have documented the presence of minor epitope variants within days of seroconversion, rising to fixation quickly in the first few weeks. However our results, presented in Fig. 1A, suggest that there was little escape happening this early on. Since reversion of escape mutations in mismatched hosts is relatively slow [23] we used the proportion of escape in HLA-mismatched hosts at first time point as an estimate of the prevalence of transmitted escape for each epitope, and hence by comparison with the proportion of escape in HLA-matched hosts obtained a quantitative estimate for the frequency of escape before the first time point. As an example, for TST (an epitope that is known to escape particularly early [39]), at the first time point 45% of HLA-mismatched patients had an escaped form of the epitope. We therefore assume that 45% of the 7 HLA-matched patients would have been infected with an escaped epitope. Hence, with reference to S5 Table, the estimated number of escape events in TST that have arisen since baseline is 6–7 × 0.45 = 2.9. Summing over all epitopes, these calculations gave the estimated ‘survival’ of wild-type epitopes in the weeks between seroconversion and first available sequence data (an average of 18.5 weeks) as 98.9% compared to 97.1% in the first 12 weeks of observation in which patients were off ART (see S1 Text for further details). Hence, whilst we have missed a handful of escape events (: 6 in total), these data do not support the idea that the majority of escape happens very early, within the first few weeks or months of infection.

The high prevalence of escape at baseline might suggest that the incidence of escape is low because many of the epitopes that are prone to acquire escape mutations already have them transmitted at baseline. This was true of the *env* epitopes we studied, which is unsurprising given the various and strong selection pressured on *env*, and a few others such as Gag TST. However, whilst there were epitopes in which the process of escape was complete even at baseline, this was not the general rule; Fig. 4 shows that more than 70% of HLA-matched patient-epitope pairs remain unescaped throughout the three-year observation period. Nor is it the case that all of the fastest escaping epitopes end up escaping in all HLA-matched patients. In these data only two epitopes (KYK and TAF) escape in all HLA-matched patients within the observation period.

The fastest rate of escape was seen within epitopes against which a positive CD8^+^ T-cell response had been measured (a group comprising: 30% of HLA-matched host-epitope pairs). Even here the incidence of escape was low (Fig. 4B). Hence we cannot conclude that it was a lack of strong CD8^+^ T-cell responses in the hosts to the set of epitopes we studied that led to few escape events being observed. However, as only IFN-γ production was measured to indicate the strength of the response we cannot tell whether or not the responses measured were qualitatively good in other ways.

Lastly, the definition of escape that we have used may have prevented some true escape from being classified as such. We were able expand the definition in two ways: by considering amino acid mutations that occur outside of known escape sites (but still within the epitope), and by extending the list of epitopes beyond the set of optimal defined epitopes (see S1 Text and S6 Table). Repeated analyses given these less stringent definitions are presented in S7–S12 Figs. Both of these amendments reduce the time to first escape in a patient so that an estimated: 10% (down from: 30%) of patients have driven no escape in HLA-restricted epitopes within the first two years. Changing the definition does not change the conclusion that the majority of escape at baseline is transmitted, or that escape mutations rise to fixation with a roughly uniform frequency across the observation period. However, extending the list of epitopes diminishes the association between HLA relative hazard and time to escape, suggesting that the strength of this association is sensitive to the set of epitopes considered.

We acknowledge that even this extended epitope list is unlikely to be complete, and that the Los Alamos ‘A-list’ will evolve to include new peptides and HLA-restrictions [40, 41]. Indeed, when using the extended list we observe 144 vs. 69 incident ‘escapes’ in HLA-mismatched vs.-matched patient-epitope pairs (S12 Fig.). Many of these ‘escapes’ in supposedly unrestricted epitopes cannot be accounted for by overlap with another, HLA-restricted epitope. Some of these may be indicative of immune escape driven by other components of the immune response such as NK cells, CD4^+^ T-cells or the antibody response. But understanding the impact of these alternative selective pressures is not necessary for assessing the concern that our methods are blind to CTL escape occurring in previously unidentified epitopes. We have previously shown that HLA-association studies favour the detection of rapid escape mutations. Hence any escape in unknown or poorly characterised epitopes that we have not looked in is likely to be slower than the time frame of this study and consequently not affect our conclusions [42].

Our definition of escape was also based on consensus viral sequence data; hence we were unable to detect the presence of any low frequency variants unlike with deep sequencing methods [2, 17]. However, variants that never rise to a majority in the population are of limited interest since they lack biological significance in terms of being unlikely to effect viral dynamics or the course of the disease in the long term. Variants that do rise to greater than 50% prevalence would be detectable with our methods. Therefore our use of consensus viral sequence is not problematic.

The final aspect of our definition of escape to address is the use of HXB2 as our comparison sequence. Although there is no ‘gold standard’ comparator for this analysis, there were only four epitopes in which HXB2 differed from the ‘A list’ optimal at a documented escape site. We therefore re-ran the analysis re-classifying these four epitopes and found no significant difference in the distribution of baseline escape prevalence between HLA-matched and-mismatched hosts. We did identify 4 new incident escapes (3 in EVK, 1 in RPN). Although the EVK escape variant is only poorly defined, its inclusion would mean that an estimated 25% of patients had not driven an escape by the end of 2 years off-therapy (as opposed to 33%).

In conclusion, this study of a cohort from a randomised controlled trial, the proper design for assessing rates of escape across a population, reveals that escape is on average both less frequent and less concentrated in the first few months of infection than would be expected from recent intense studies of much smaller numbers of acutely infected individuals.

## Materials and Methods

### Ethics statement

The SPARTAC trial was approved by the following authorities: Medicines and Healthcare products Regulatory Agency (UK), Ministry of Health (Brazil), Irish Medicines Board (Ireland), Medicines Control Council (South Africa), and The Uganda National Council for Science and Technology (Uganda). It was also approved by the following ethics committees in the participating countries: Central London Research Ethics Committee (UK), Hospital Universitrio Clementino Fraga Filho Ethics in Research Committee (Brazil), Clinical Research and Ethics Committee of Hospital Clinic in the province of Barcelona, Spain, The Adelaide and Meath Hospital Research Ethics Committee (Ireland), University of Witwatersrand Human Research Ethics Committee, University of Kwazulu-Natal Research Ethics Committee and University of Cape Town Research Ethics Committee (South Africa), Uganda Virus Research Institute Science and ethics committee (Uganda), The Prince Charles Hospital Human Research Ethics Committee and St Vincent’s Hospital Human Research Ethics Committee (Australia), and the National Institute for Infectious Diseases Lazzaro Spallanzani, Institute Hospital and the Medical Research Ethics Committee, and the ethical committee Of the Central Foundation of San Raffaele, MonteTabor (Italy). All participants signed a written informed consent.

### Participants and trial design

The design of the SPARTAC trial is reported elsewhere [32]. In brief, SPARTAC was an international open Randomised Controlled Trial enrolling adults with PHI within 6 months of a last negative, equivocal or incident HIV-1 test. All participants gave written informed consent. The trial was approved by research ethics committees in each country. Time of seroconversion was estimated as the midpoint of last negative/equivocal and first positive tests, or date of incident test. Participants were randomised to receive ART for 48 weeks (ART-48), 12 weeks (ART-12) or no therapy (standard of care, SOC). The primary endpoint was a composite of two events: if participants either reached a CD4 count of <350 cells/mm^3^ (> 3 months after randomisation and confirmed within 4 weeks) or initiated long-term ART. This provided an immunological surrogate of clinical progression, but also allowed inclusion of those participants who commenced ART at CD4 cell counts greater than cells/mm^3^.

The participants studied here consisted of a sub-group 125 HIV-1 subtype B positive individuals of whom 42, 41 and 42 were randomised to ART-12, ART-48 and SOC, respectively. Peripheral blood mononuclear cells (PBMCs) were collected at regular intervals during the treatment period and follow-up. The sub-group of 125 participants was based on those individuals infected with subtype B HIV-1 for whom both sequence and ELISpot data were available, predominantly determined by specimen availability.

### Sequences

Viral RNA was extracted from patient plasma (Qiagen Viral RNA extraction kit) and the HIV-1 *pol*, *gag*, *nef* and *env* genes were amplified separately using nested PCR reactions and primers described previously [20, 37, 43]. The PCR products were purified and sequenced using Big Dye dideoxy terminator chemistry (ABI). Sequences are therefore presented as the consensus from a bulk PCR product for each time-point. Sequence data were available for each patient at a subset of the following time points: 0, 16 or 24, 52 or 60, 108 and 156 weeks following recruitment onto the trial (see S1 Table for details).

### CD8 T cell ELISpot assays

Quantification of CD8^+^ T-cell response was performed by interferon gamma ELISpot analysis in all subtype B patients, using methods described elsewhere [44]. Responses were determined to 181 subtype B optimal peptides covering the Gag (n = 67), Pol (n = 47), Nef (n = 30) and Env (n = 37) proteins and 195 autologous variants synthesised followed sequence analysis. The optimal peptides tested were derived from the “best-defined ‘A list’ CTL epitope” database accessed from the Los Alamos HIV Immunology Website. Analyses were performed singly, but in duplicate for the negative control, and results expressed as spot forming units (SFU) per 10^6^ cells. ELISpot responses were measured at 0, 24 and 60 weeks post-recruitment.

### HLA genotyping

Patients’ HLA type was determined to the oligo-allelic level using Dynal RELITM Reverse Sequence-Specific Oligonucleotide kits for the HLA-A, -B and -C loci (Dynal Biotech). To obtain four-digit typing, Dynal Biotech Sequence-Specific priming kits were used, in conjunction with the Sequence-Specific Oligonucleotide type.

### Defining escape

A CTL escape mutation was defined as *any amino acid variation in previously identified (and phenotypically confirmed) escape sites within an* ‘*optimal’ defined epitope*. Known escape sites were defined as within-epitope sites for which escape (E), calculated escape (CE), inferred escape (IE) or literature escape (LE) had been documented in the ‘CTL/CD8^+^ Epitope Variants and Escape Mutations’ HIV database table, as downloaded on 05/11/12 [45]. Any amino acid difference between autologous sequence and HXB2 in one of the sites under consideration was classed as escape. The list of ‘optimal’ defined epitopes was initially drawn from the set of epitopes for which ELISpot assays had been conducted (see above) though this was later extended (S1 Text and S6 Table). The 4-digit HLA-type restricting each epitope was determined using the ‘Best-defined’ epitope summary table on the Los Alamos HIV Immunology Database [45] downloaded on 01/03/2013. 2-digit HLA types were used only where 4-digit HLA types were not available. Only epitopes restricted by HLA-A and-B alleles were included since data on patient HLA-C alleles was not initially available. However a subsequent analysis found there were no incident escape mutations observed in any of these patients in epitopes restricted by their HLA-C alleles, so the exclusion of these epitopes is not problematic.

This intentionally stringent definition of escape was chosen to optimise the balance between including all relevant variation available in sequence data whilst excluding variation that is not escape. This definition does not include escape mutations that lie outside the epitope and disrupt proteasomal processing or antigen presentation pathways. It also excludes escape that occurs in poorly studied or unknown epitopes [46]. To complement the published data available and to check that mutations classed as ‘escape’ genuinely did result in diminished IFN-γ production by CD8^+^ T-cells, a comparison was made between the magnitudes of CD8^+^ T-cell ELISpot responses for the pre-defined optimal peptides with those for autologous epitope variants observed in the population at baseline. Variants for 7 Gag and 2 Pol epitopes were tested. In all cases except Gag EVD (where the optimal peptide differed from the HXB2 sequence) the major variant(s) elicited a diminished response. In 6/9 cases responses to all ‘escape’ variants were reduced by at least 90% (Table S2).

### Identification of escape in the data

Firstly, the sequence data was subjected to quality control: A phylogenetic tree was constructed for all sequences of each gene in turn so that patients whose sequences did not cluster with each other could be identified and removed from the dataset if necessary. Due to difficulties in aligning all regions of all genes, epitopes were located using a string-matching search, allowing for 4 amino acid differences between the HXB2 epitope sequence and the patient sequence. Spurious matches were identified by hand and removed. Missing data resulted in escape being classed as ‘not determined’ if the HXB2 and viral sequence matched at all of the defined escape sites in the epitope except those where data was missing.

### Statistics

**Permutation test on the distribution of escape between HLA-matched and-mismatched hosts**. We wished to test the null hypothesis that escape is equally likely to be present in HLA-matched and-mismatched hosts against the alternative hypothesis that there is more escape in HLA-matched hosts. The statistic used for this was the mean (across all epitopes) of the Fisher’s exact test p-values for whether escape was independent of HLA-matching for that epitope. A permutation test was used to calculate the distribution of this mean under the null hypothesis; 2,000 random data sets were generated by permuting which patients the escape events occurred in for each epitope (i.e. there was no permutation between epitopes). The mean of the p-values for the observed data was then calculated and compared to the null distribution (S1 Fig.).

**Poisson process for calculation of expected escape distributions**. For each epitope in each patient in which an incident escape could have been observed, the total amount of time for which the patient was observed whilst off ART was calculated. Let E be the total number of escape events observed in the population, T be the total observation time (whilst off ART) across all epitopes in all patients and t_p_ be the total observation time for all epitopes of a particular patient, p. Then, under the assumption that the average rate of incidence of escape in all patients is r = E/T, the probability of patient p driving i escape events is:

$$\text{Pr}(\;p\;\text{drives}\;i\;\text{escape events during observation})=\frac{e^{-t_{p}r}{\left(t_{p}r\right)}^{i}}{i!}$$

The expected number of patients with i incident escape events is then the sum over all patients of these probabilities. The expected distribution of escape across different epitopes is calculated similarly.

To account for missing data (for Fig. 2B), it was supposed that each patient had 108 weeks of data, during which time they were off ART. Where an epitope sequence was entirely missing (so that whether or not the epitope was initially wild-type could not be determined), the probability of it being wild-type at baseline was taken to be equal to the prevalence of escape at the first time point, as in S5 Table (this was necessary for a total of 203 HLA-matched patient-epitope pairs). We could then calculate *t*_*p*_ = (#HLA-matched, initially wild-type epitopes)×108.

Note that this model does not impose the condition that only one escape is possible in each patient-epitope pair. We believe this to be a reasonable simplification given that the total number of escape events is small.

A X^2^ goodness-of-fit test with 4 degrees of freedom (i.e. categories for 0, 1, 2, 3, and > 4 escaped epitopes) was used to test the null hypothesis that the observed data followed the expected distribution.

**Survival analysis**. For the survival analysis shown in Figs. 3 and 4 only patient-epitope pairs in which it would have been possible to observe an escape were considered. This included all patient-epitope pairs that were wild-type in known escape sites at the first data point and had data available at a second data point. For each patient-epitope pair where an incident escape occurred, escape times were estimated as the midpoint between the last wild-type observation (or if that observation was during therapy, the therapy end time) and the first appearance of the escape mutant in the consensus sequence (or the clinical endpoint, whichever was earlier). Escape events occurring during treatment were discarded. Where no incident escape occurred, right-censoring started at the last sequence data time point or clinical endpoint, whichever was earlier. In Fig. 3, hazard ratios were calculated using a simple Cox Proportional Hazards model with a single predictor—the variable of interest. p-values relate to the Likelihood Ratio test comparing the one-predictor model with the null model. In Fig. 4, where observations from all patients and epitopes were pooled, hazard ratios and associated p-values were calculated using a mixed effects Cox model with a single predictor but also random intercept terms for patient and epitope ID. This was implemented with the package ‘coxme’ in R.

## Supporting Information

S1 Text Supplementary methods describing estimation of number of escape events before the first time point, analysis of whether censoring from the survival analysis is independent of escape, multivariate Cox models for time to escape and extending the definition of escape. (PDF)

S1 Fig The distribution of escape between HLA-matched and mismatched host-epitope pairs at baseline and 1 year later. Histograms show the distribution of the mean of the Fisher’s exact test p-values for independence of HLA-matching and escape in each epitope, under the null hypothesis that ‘escape’ is equally likely to be seen in HLA-matched and-mismatched hosts. (A) at baseline, (B) at 52 weeks. The red lines show the observed mean of the p-values. 2000 random simulations were used to obtain the null distributions. There is significant evidence to reject the null hypothesis at 52 weeks but not baseline, suggesting that little escape has already been driven before baseline.
(EPS)

S2 Fig Defining the relationship between clinical failure and censoring. Each patient-epitope pair was categorised according to if and when the patient reached the trial endpoint and how their time of clinical failure related to the last available sequence data point, as shown here diagrammatically. The three categories are (A) censoring may have resulted from clinical failure and there were no observations after failure, (B) censoring may have resulted from clinical failure but there was at least 1 further observation after failure, and (C) censoring is not related to clinical failure since long course ART was started after the last observed data point. Numbers on the right hand side represent the number of HLA-matched patient-epitope pairs and in brackets the number of distinct patients in each category. Note that three patients have epitopes in both A and B. One patient was removed as they were suspected to have been superinfected during the observation period.
(EPS)

S3 Fig Kaplan-Meier survival cure for wild-type epitopes, split by restricting HLA. All HLA-matched host-epitope pairs are considered. Epitopes restricted by a protective HLA-allele as defined in Fig. 3 (light red) escape sooner than others (dark red). There is no significant increased risk associated with restriction by a protective allele here (p = 0.3, mixed effects Cox model as in Fig. 4), though narrowing the definition of ‘protective’ does result in significance.
(EPS)

S4 Fig Kaplan-Meier plots to show time to first escape (using midpoint approximation) within the patients of this cohort, split by the duration of therapy they have received. Patients who were either missing data for *gag* or *nef*, the two genes for which the most data was available, were not included so as not to skew the results due to lack of data. Time to first escape in an HLA-restricted epitope is plotted. These survival curves are very similar for the patients who have received no ART and who have received < 6 months of ART. Patients who have received > 6 mo ART were observed for less time off ART and hence the number of patients in this group with enough data to be included in the analysis is smaller (15 patients compared to 22 with no ART and 24 with < 6 mo ART). Therefore, while there appears to be a slower rate of escape in patients who have received > 6 months ART this difference is not significant (p = 0.24) and the effect of removing this group from the data is small (see S5 Fig.).
(EPS)

S5 Fig Kaplan-Meier plots to show time to first escape (using midpoint approximation) within the patients of this cohort, excluding patients who have received > 6 mo ART. Patients who were either missing data for *gag* or *nef*, the two genes for which the most data was available, were not included so as not to skew the results due to lack of data. (A) Time to first escape in an HLA-restricted epitope (solid line) is plotted along with 95% confidence intervals (dotted line). (B) Patients are split according to whether they have one of the more ‘protective’ HLA alleles or not. The set of beneficial alleles was taken to be B*58, B*27, B*57, A*26, B*51, A*11, B*14, B*18, B*08 (all the HLA-A and-B alleles down to B*08 that are present in our data, taken from the ranking in [47]). Having a protective HLA resulted in a significantly increased risk of escape in an HLA-restricted epitope (p = 0.03, HR = 3.3). In both plots the x-axis represents the time since cessation of ART, or baseline for those who did not receive ART, and vertical checks mark time points at which patients were censored (either because they began long term ART or because there was no further sequence data available for them). Numbers indicate the number of patients who have not yet escaped or been censored at the corresponding red dots (time points: 0, 27, 55, 93 wks).
(EPS)

S6 Fig Kaplan-Meier survival curves for wild-type epitopes, excluding patients who have received > 6 mo ART. As with time to first escape in a patient, time to escape in epitopes was longer for epitopes in patients who have previously received > 6 mo ART, but there was no difference between epitopes in those who had received no ART and in those who had received < 6 mo ART. (A) Time to a first escape in epitopes within HLA-mismatched (dark blue) and-matched (light blue) hosts is shown. (B) Epitopes in HLA-matched hosts are split according to whether they have a measurable CD8^+^ T-cell ELISpot response to them (light red) or not (dark red). As in Fig. 4, escape is strongly significantly faster within epitopes in HLA-matched hosts (p < 1×10^-4^, HR = 2.5) and then faster again if the host has a measurable CD8^+^ T-cell ELISpot response to that epitope (p = 0.034, HR = 2.7). p-values and hazard ratios were calculated using a mixed effects model as in Fig. 4.
(EPS)

S7 Fig Escape prevalence in HLA-matched and-mismatched hosts, where escape is defined as any mutation within the epitope. Epitopes are labelled where possible with their first three amino acids. Bars represent 95% binomial confidence limits (Agresti-Coull method). The dotted black line is y = x. Epitopes labelled in bold have a significantly higher proportion of escape in matched hosts than in mismatched hosts (one-tailed Fisher’s Exact test, p < 0.1). Note that this plot is noisier that Fig. 1 since a smaller proportion of the mutations classified as ‘escape’ are likely to be CTL related. Consequently the distribution at neither time point is significantly different from that under the null hypothesis that escape is equally likely to occur in HLA-matched and-mismatched hosts (permutation test, see Methods). (A) At baseline the majority of epitopes have escape at equal prevalence in HLA-matched and-mismatched hosts (B) 52 weeks later within host evolution has resulted in a higher prevalence of escape in HLA-matched hosts for many epitopes, with six lying significantly ‘below the line’ despite a decrease in the number of patients with sequence data available at 52 weeks.
(EPS)

S8 Fig Kaplan-Meier plots to show time to first escape (using midpoint approximation) within the patients of this cohort, excluding patients who have received > 6 mo ART, where escape is defined as any mutation within the epitope. Patients who were either missing data for *gag* or *nef*, the two genes for which the most data was available, were not included so as not to skew the results due to lack of data. (A) Time to first escape in an HLA-restricted epitope (solid line) is plotted along with 95% confidence intervals (dotted line). (B) Patients are split according to whether they have one of the more ‘protective’ HLA alleles or not. The set of beneficial alleles was taken to be all the HLA-A and-B alleles down to B*08 that are present in our data, taken from the ranking in [47]. Having a protective HLA resulted in a significantly increased risk of escape in an HLA-restricted epitope (p = 0.003, HR = 3.6). In both plots the x-axis represents the time since cessation of ART, or baseline for those who did not receive ART, and vertical checks mark time points at which patients were censored (either because they began long term ART or because there was no further sequence data available for them). Numbers indicate the number of patients who have not yet escaped or been censored at the corresponding red dots (time points: 0, 27, 55 wks).
(EPS)

S9 Fig Kaplan-Meier survival curves for wild-type epitopes, excluding patients who have received > 6 mo ART, where escape is defined as any mutation within the epitope. (A) Time to a first escape for epitopes in HLA-mismatched (dark blue) and-matched (light blue) hosts is shown. (B) Epitopes within HLA-matched hosts are split according to whether they have a measurable CD8^+^ T-cell ELISpot response to them (light red) or not (dark red). As in Fig. 4, escape is strongly significantly faster within epitopes in HLA-matched hosts (p < 1×10^-4^, HR = 2.2) and then faster again if the host has a measurable CD8^+^ T-cell ELISpot response to that epitope (p = 0.001, HR = 2.9). p-values and hazard ratios were calculated using a mixed effects model as in Fig. 4.
(EPS)

S10 Fig Escape prevalence in HLA-matched and-mismatched hosts, where the escape list has been extended beyond the optimals to all epitopes < 12 aa in length with a 4-digit HLA restriction defined. Epitopes are labelled where possible with their first three amino acids. Bars represent 95% binomial confidence limits (Agresti-Coull method). The dotted black line is y = x. Epitopes labelled in bold have a significantly higher proportion of escape in matched hosts than in mismatched hosts (one-tailed Fisher’s Exact test, p < 0.1). (A) At baseline the majority of epitopes have escape at equal prevalence in HLA-matched and-mismatched hosts and the distribution of points is not significantly different from as would be expected if escape was equally likely to occur in HLA-matched and-mismatched hosts (p = 0.5, permutation test) (B) 52 weeks later within host evolution has resulted in a higher prevalence of escape in HLA-matched hosts for many epitopes, with the eight epitopes and the distribution as a whole lying significantly ‘below the line’ (p < 0.003, permutation test, see Methods).
(EPS)

S11 Fig Kaplan-Meier plots to show time to first escape (using midpoint approximation) within the patients of this cohort, excluding patients who have received > 6 mo ART, where the escape list has been extended beyond the optimals to all epitopes < 12 aa in length with a 4-digit HLA restriction defined. Patients who were either missing data for *gag* or *nef*, the two genes for which the most data was available, were not included so as not to skew the results due to lack of data. (A) Time to first escape in an HLA-restricted epitope (solid line) is plotted along with 95% confidence intervals (dotted line). (B) Patients are split according to whether they have one of the more ‘protective’ HLA alleles or not. The set of beneficial alleles was taken to be all the HLA-A and-B alleles down to B*08 that are present in our data, taken from the ranking in [47]. Having a protective HLA resulted in an increased risk of escape in an HLA-restricted epitope early on, but this was not significant. In both plots the x-axis represents the time since cessation of ART, or baseline for those who did not receive ART, and vertical checks mark time points at which patients were censored (either because they began long term ART or because there was no further sequence data available for them). Numbers indicate the number of patients who have not yet escaped or been censored at the corresponding time points marked by red dots 0, 27, 55 wks.
(EPS)

S12 Fig Kaplan-Meier survival curves for wild-type, epitopes excluding patients who have received > 6 mo ART, where the escape list has been extended beyond the optimals to all epitopes < 12 aa in length with a 4-digit HLA restriction defined. Time to a first escape for epitopes in HLA-mismatched (dark blue) and-matched (light blue) hosts is shown. As in Fig. 4, escape is strongly significantly faster within epitopes in HLA-matched hosts (p < 1×10^-10^, HR = 3.2). The effect of a response could not be analysed as CD8^+^ T-cell ELISpot responses were not measured for all of the extended list of epitopes. The p-value and hazard ratio was calculated using a mixed effects model as in Fig. 4.
(EPS)

S1 Table The number of individual patients for which viral sequence and CD8^+^ T-cell ELISpot data is available. Note that some sequences do not cover the whole gene and so the number given is an upper bound for the number of patients who have sequence data for any one epitope. For each patient ELISpot data was either available for all 181 optimal peptides across all four genes or not at all. Data was available in 115 patients at week 0, 109 patients at week 24 and 115 patients at week 60. The final column gives the number of patients with at least 2 time points of sequence data and ELISpot data at at least one of 0, 24 or 60 weeks.
(PDF)

S2 Table Diminished responses to variants present in the population at baseline. All epitopes for which CD8^+^ T-cell responses to autologous variants were assessed and for which there was at least one HLA-matched patient with a positive response to the optimal epitope are shown. The top sequence for each epitope gives the pre-defined optimal ELISpot peptide, which is also the most prevalent variant in each case. The ‘Escape’ column records whether or not each variant is classified as escape according to our definition. The prevalence of each variant in the cohort at baseline and the proportion of patients with a particular variant who are HLA-matched for the epitope are also recorded. The final column gives the average ELISpot response to the variant (number of SFCs) in those that are HLA-matched and respond to the optimal, as a percentage of their response to the optimal.
(PDF)

S3 Table The numbers of HLA-matched and-mismatched patients with escape at baseline. All p-values < 0.2 are given for a Fisher’s exact test with the alternative hypothesis that the proportion of escape in matched hosts is greater than that in mismatched hosts.
(PDF)

S4 Table The numbers of HLA-matched and-mismatched patients with escape after 1 year. All p-values < 0.2 are given for a Fisher’s exact test with the alternative hypothesis that the proportion of escape in matched hosts is greater than that in mismatched hosts.
(PDF)

S5 Table The numbers of HLA-matched and-mismatched patients with escape at the first time point. Since a few patients did not have available sequence data at baseline, this table is similar but not identical to S3 Table. The number of incident escape events observed within HLA-matched and-mismatched patients is also given.
(PDF)

S6 Table All epitopes in first extended definition of escape. Peptides that are also in the ‘A-list’ of optimal epitopes are shown in bold.
(PDF)
