# Supplementary material for: Structured Observations Reveal Slow HIV-1 CTL Escape
Source: PLoS Genet. 2015 Feb 2;11(2):e1004914. doi: 10.1371/journal.pgen.1004914 (PMC4333731; doi:10.1371/journal.pgen.1004914)
Supplement: S1 Text — (PDF) [file pgen.1004914.s001.pdf]

Structured observations reveal slow HIV-1 CTL escape

Text S1: Supplementary Methods

Estimating the number of escape events before first time point

The data in Table S5 was used for this analysis. The prevalence of escape in HLA-mismatched hosts was used as an estimate for the true prevalence of transmitted escape so that an estimate for the number of transmitted escaped epitopes in HLA-matched hosts could be inferred. We then calculated the number of escape events driven before baseline as

$$\sum_{\text{epitopes}} (\text{HLA-matched patients with escape at first time point} - \text{HLA matched patients with transmitted escape})$$

giving a total of 5.9 escape events in epitopes in HLA-matched hosts. Since there were 530 HLA-matched patient-epitope pairs that were wild-type at the first time point this is 5.9 out of a possible $530 + 5.9 = 535.9$ (the estimated number of wild-type epitopes in HLA-matched hosts at infection). The mean time between seroconversion and first available sequence data across all patient-epitope pairs was found to be 18.5 weeks. Therefore $(535.9 - 5.9)/535.9$ gives a survival percentage of 98.9% in the first 18.5 weeks.

Is escape associated with clinical failure and if so would this lead us to underestimate the rate of escape?

In the 'time to escape' analysis of Figures 3 and 4, patients are censored from the survival analysis if they have no further sequence data or if they start long term ART (usually because they have reached the primary endpoint of the trial). If escape plays a part in driving clinical failure then those patients that were censored due to clinical failure during the three-year observation period may have had high rates of escape that we were unable to observe, leading us to underestimate the rate of escape.

We initially split all HLA-matched patient-epitope pairs into those that were censored from the survival analysis as a result of clinical failure within the three-year observation period and those whose censoring was unrelated to clinical failure (Fig S2C). This first group was further divided into those whose

last sequence data sample was before clinical failure (Fig S2A), and those whose last sample was after clinical failure (Fig S2B). Most of the patient-epitope pairs fell in category C.

The escape events that we are concerned we may have been unable to observe are those that would have been driven soon before clinical failure but remained unobserved since the patient had no further sequence data after failure. There are 20 and 24 patients with final sequence data before and after clinical failure respectively, and incident escape events were observed in 6 and 7 of these patients resp. (Fig S2). Examination of the patients in category B showed that 75 patient-epitope pairs were wild-type at the time point before clinical failure and of these only two were observed to escape. Hence only two incident escape events would have been missed had sequence data not been available at the post- clinical failure time point. Given the similar numbers of patients in groups A and B it is fair to assume that the number of escape events that went unobserved in patients that were censored due to clinical failure (in A) was also around two. Therefore the extent to which escape is linked to censoring time is small and we do not believe that patients being censored after clinical failure has lead us to underestimate the rate of escape.

Multivariate Cox models for time to escape

In order to see whether the association between HLA relative risk and time to escape persisted when other factors were taken into account that could be confounding, we made a multivariate Cox proportional hazards model. We included predictors for time spent on ART, baseline viral load (VL), baseline CD4 count, number of responses each patient made to wild-type epitopes at baseline, and whether or not the patient had a protective HLA type. The HLA predictor remained in the model along with baseline viral load and CD4 count, but neither VL nor CD4 count were associated with a non-negligible hazard ratio, hence the association between possession of a protective HLA allele and time to first escape seems genuine.

Extending the definition of escape

The first amendment to the definition of escape considers all within-epitope mutations as 'escape' and so the epitope list is also no longer restricted to include only those epitopes with phenotypically verified escape sites. The resulting list in Table S6 is the intersection of the HIV database 'A-list' of best defined epitopes and the set of optimal epitopes for which ELISpot assays were conducted.

The second amendment extended the epitope list so that it was not restricted to peptides for which

ELISpot assays had been performed and also included epitopes on the longer list of epitopes available from the LANL immunology database [45]. Where a 'best-defined' epitope HLA restriction was available it was used.
