# Supplementary material for: Structured Observations Reveal Slow HIV-1 CTL Escape
Source: PLoS Genet. 2015 Feb 2;11(2):e1004914. doi: 10.1371/journal.pgen.1004914 (PMC4333731; doi:10.1371/journal.pgen.1004914)
Supplement: S1 Table — Note that some sequences do not cover the whole gene and so the number given is an upper bound for the number of patients who have sequence data for any one epitope. For each patient ELISpot data was either available for all 181 optimal peptides across all four genes or not at all. Data was available in 115 patients at week 0, 109 patients at week 24 and 115 patients at week 60. The final column gives the number of patients with at least 2 time points of sequence data and ELISpot data at at least one of 0, 24 or 60 weeks. (PDF) [file pgen.1004914.s014.pdf]

Gene	Week (approximate)	Number of UK patients with data for viral sequence	2 t.p.s + ELISpot
gag	0	89	83
	24	60	
	52	66	
	108	35	
	156	8	
pol	0	96	54
	16 or 24	30	
	52 or 60	41	
	108	17	
	156	1	
env	0	70	55
	24	38	
	52	51	
	108	0	
	156	0	
nef	0	76	54
	24	60	
	52	62	
	108	39	
	156	12	

Table S1
