# Supplementary material for: Structured Observations Reveal Slow HIV-1 CTL Escape
Source: PLoS Genet. 2015 Feb 2;11(2):e1004914. doi: 10.1371/journal.pgen.1004914 (PMC4333731; doi:10.1371/journal.pgen.1004914)
Supplement: S2 Table — All epitopes for which CD8+ T-cell responses to autologous variants were assessed and for which there was at least one HLA-matched patient with a positive response to the optimal epitope are shown. The top sequence for each epitope gives the pre-defined optimal ELISpot peptide, which is also the most prevalent variant in each case. The ‘Escape’ column records whether or not each variant is classified as escape according to our definition. The prevalence of each variant in the cohort at baseline and the proportion of patients with a particular variant who are HLA-matched for the epitope are also recorded. The final column gives the average ELISpot response to the variant (number of SFCs) in those that are HLA-matched and respond to the optimal, as a percentage of their response to the optimal. (PDF) [file pgen.1004914.s015.pdf]

Epitope	Escape?	Prevalence	HLA matched	ELISpot response (%)
S L Y N T V A T L	HXB2	28/84	0.68	100.0
- - - - - I - V -	Y	16/84	0.69	69.6
- - F - - - - - -	Y	12/84	0.42	0.0
- - - - - - - V -	Y	12/84	0.50	1.1
- - F - - - - V -	Y	9/84	0.56	31.6
- - F - A - - V -	Y	2/84	1.00	0.0
- - F - - I - V -	Y	2/84	1.00	31.6
- - - - - I - - -	Y	2/84	1.00	115.2
- - F - - I - - -	Y	1/84	0.00	0.0
R L R P G G K K K	HXB2	45/75	0.09	100.0
- - - - - - - - Q	Y	16/75	0.06	0.0
- - - - - - - - R	Y	5/75	0.20	0.0
- - - - - - R - -	Y	5/75	0.00	5.1
- - - - - - - - T	Y	3/75	0.00	0.0
- - - - - - - - N	Y	1/75	1.00	0.0
A C Q G V G G P G H K	HXB2	70/84	0.09	100.0
- - - - - - - - S - -	Y	14/84	0.07	0.0
K R W I I L G L N K	HXB2	67/81	0.10	100.0
- - - - - M - - - -	Y	11/81	0.09	0.0
- K - - - M - - - -	Y	3/81	0.00	0.0
N A N P D C K T I	HXB2	69/84	0.06	100.0
- S - - - - - - -	Y	13/84	0.08	0.0
- - - - - - - I -	Y	2/84	0.00	0.0
E V K D T K E A L	Y	25/83	0.36	100.0
D - - - - - - - -	Y	24/83	0.13	45.4
- I - - - - - - -	HXB2	14/83	0.36	62.8
D - R - - - - - -	Y	11/83	0.18	0.0
- I R - - - - - -	N	4/83	0.25	0.0
D I - - - - - - -	Y	3/83	0.00	41.9
- - Q - - - - - -	Y	2/83	0.50	0.0
I L K E P V H G V	HXB2	77/89	0.57	100.0
- - - - - - - - A	Y	7/89	0.57	0.0
- - R - - - - - -	Y	5/89	0.60	0.0
T S T L Q E Q I G W	HXB2	43/73	0.00	100.0
- - - - - - - - A -	Y	20/73	0.05	36.9
- - N - - - - - A -	Y	6/73	0.33	5.7
- - N - - - - - T -	Y	2/73	0.50	7.7
- - - - - - - - D -	Y	1/73	1.00	55.7
- - - - - - - - E -	Y	1/73	1.00	54.0
T A F T I P S I	HXB2	50/83	0.02	100.0
- - - - - - - T	Y	33/83	0.06	0.6

Table S2
