# Supplementary material for: Structured Observations Reveal Slow HIV-1 CTL Escape
Source: PLoS Genet. 2015 Feb 2;11(2):e1004914. doi: 10.1371/journal.pgen.1004914 (PMC4333731; doi:10.1371/journal.pgen.1004914)
Supplement: S3 Table — All p-values < 0.2 are given for a Fisher’s exact test with the alternative hypothesis that the proportion of escape in matched hosts is greater than that in mismatched hosts. (PDF) [file pgen.1004914.s016.pdf]

Epitope	HLA restriction	Matched	with escape	Mismatched	with escape	p
ACQGVGGPGHK	A*1101	7	1	78	13	
AENLWVTVY	B*1801	2	2	67	61	
AIFQSSMTK	A*0301, A*1101	23	0	72	6	
AVDLSHFLK	A*0301, A*1101	13	1	62	6	
DCKTILKAL	B*0801	20	1	65	0	
DRFYKTLRA	B*1402	5	0	80	5	
EIYKRWII	B*0801	20	3	65	8	
ELRSLYNTV	B*0801	21	14	67	51	
EVIPMFSAL	A*2601	5	2	83	25	
EVKDTKEAL	B*0801	21	15	67	54	
FLKEKGGL	B*0801	19	3	56	9	
GEIYKRWII	B*0801	20	3	64	7	
GELDRWEKI	B*4002	2	0	85	2	
GGKKKYKLK	B*0801	21	4	67	9	
HTQGYFPDWQ	B*5701	5	1	71	18	
ILKEPVHGV	A*0201	54	9	41	7	
ILKEPVHGVY	B*1501	14	1	81	8	
IRLRPGGKK	B*2705	9	2	79	11	
ISPRTLNAW	B*5701	7	2	81	27	
IVLPEKDSW	B*5701	8	5	83	32	0.17
KAFSPEVIPMF	B*5701, B*5703	7	0	81	1	
KEKGGLEGL	B*4001, B*4002	15	5	60	9	0.11
KIRLRPGGK	A*0301	7	1	81	18	
KRWIILGLNK	B*2705	9	2	77	16	
KYKLKHIVW	A*2402	15	11	73	59	
LVGPTPVNI	A*0201	53	0	42	1	
NANPDCKTI	B*5101	5	1	80	15	
QASQEVKNW	B*5301, B*5701	8	4	77	33	
QVPLRPMTYK	A*0301, A*1101	13	2	62	17	
RLRPGGKKK	A*0301	7	3	81	38	
RPNNNTRKSI	B*0702	10	10	59	59	
RPQVPLRPM	B*4201	0	0	75	3	
SFNCGGEFF	B*1516	0	0	70	9	
SLYNTVATL	A*0201, A*0202, A*0205	52	33	36	27	
TAFTIPSI	B*5101	4	3	90	39	
TPGPGVRYPL	B*0702,B*4201	15	0	61	3	
TPQDLNTML	B*0702, B*3910, B*4201, B*8101	14	0	73	2	
TSTLQEQIGW	B*5701, B*5801	6	6	80	36	0.01
VIYQYMDDL	A*0201	54	2	42	3	
VLEWRFDSRL	A*0201	43	17	29	14	
VPLRPMTY	B*3501	5	1	69	13	
WPTVRERM	B*0801	19	19	53	42	0.03
WRFDSRLAF	B*1503	2	1	71	33	
YETEVHNVW	B*1801	2	2	68	37	
YLKDQQLL	B*0801	18	18	52	49	
YPGIKVRQL	B*4201	0	0	96	90	
Totals		679	210	3152	1025	

Table S3
