# Supplementary material for: Structured Observations Reveal Slow HIV-1 CTL Escape
Source: PLoS Genet. 2015 Feb 2;11(2):e1004914. doi: 10.1371/journal.pgen.1004914 (PMC4333731; doi:10.1371/journal.pgen.1004914)
Supplement: S4 Table — All p-values < 0.2 are given for a Fisher’s exact test with the alternative hypothesis that the proportion of escape in matched hosts is greater than that in mismatched hosts. (PDF) [file pgen.1004914.s017.pdf]

Epitope	HLA restriction	Matched	with escape	Mismatched	with escape	p
ACQGVGGPGHK	A*1101	6	1	59	14	
AENLWVTVY	B*1801	1	1	50	45	
AIFQSSMTK	A*0301, A*1101	13	1	27	4	
AVDLSHFLK	A*0301, A*1101	12	4	50	3	0.02
DCKTILKAL	B*0801	15	0	50	0	
DRFYKTLRA	B*1402	4	0	61	4	
EIYKRWII	B*0801	15	3	50	6	
ELRSLYNTV	B*0801	15	11	48	38	
EVIPMFSAL	A*2601	3	2	63	15	0.16
EVKDTKEAL	B*0801	15	10	48	39	
FLKEKGGL	B*0801	16	4	46	6	
GEIYKRWII	B*0801	15	3	50	6	
GELDRWEKI	B*4002	2	0	59	0	
GGKKKYKLK	B*0801	15	2	47	6	
HTQGYFPDWQ	B*5701	5	3	57	17	0.19
ILKEPVHGV	A*0201	23	4	18	0	0.09
ILKEPVHGVY	B*1501	4	0	36	1	
IRLRPGGKK	B*2705	4	1	58	7	
ISPRTLNAW	B*5701	6	2	59	20	
IVLPEKDSW	B*5701	2	2	34	11	0.12
KAFSPEVIPMF	B*5701, B*5703	6	0	60	2	
KEKGGLEGL	B*4001, B*4002	13	3	49	6	
KIRLRPGGK	A*0301	6	2	56	12	
KRWIILGLNK	B*2705	5	2	60	12	
KYKLKHIVW	A*2402	10	9	52	42	
LVGPTPVNI	A*0201	21	0	17	1	
NANPDCKTI	B*5101	3	1	62	10	
QASQEVKNW	B*5301, B*5701	8	4	57	32	
QVPLRPMTYK	A*0301, A*1101	12	4	50	10	
RLRPGGKKK	A*0301	6	5	56	22	0.05
RPNNNTRKSI	B*0702	6	6	44	43	
RPQVPLRPM	B*4201	0	0	62	3	
SFNCGGEFF	B*1516	0	0	50	9	
SLYNTVATL	A*0201, A*0202, A*0205	34	22	29	22	
TAFTIPSI	B*5101	2	2	36	11	0.11
TPGPGVRYPL	B*0702, B*4201	12	0	50	2	
TPQDLNTML	B*0702, B*3910, B*4201, B*8101	11	0	55	3	
TSTLQEQIGW	B*5701, B*5801	6	4	60	28	
VIYQYMDDL	A*0201	23	1	18	0	
VLEWRFDSRL	A*0201	36	13	24	12	
VPLRPMTY	B*3501	6	3	56	8	0.06
WPTVRERM	B*0801	16	15	42	35	
WRFDSRLAF	B*1503	1	0	58	24	
YETEVHNVW	B*1801	1	1	50	27	
YLKDQQLL	B*0801	14	14	37	36	
YPGIKVRQL	B*4201	0	0	41	40	
Totals		449	165	2201	694	

Table S4
