# Supplementary material for: Structured Observations Reveal Slow HIV-1 CTL Escape
Source: PLoS Genet. 2015 Feb 2;11(2):e1004914. doi: 10.1371/journal.pgen.1004914 (PMC4333731; doi:10.1371/journal.pgen.1004914)
Supplement: S5 Table — Since a few patients did not have available sequence data at baseline, this table is similar but not identical to S3 Table. The number of incident escape events observed within HLA-matched and-mismatched patients is also given. (PDF) [file pgen.1004914.s018.pdf]

Epitope	HLA restriction	Matched (escaped)	Mismatched (escaped)	Incident escape	
				Matched	Mismatched
ACQGVGGPGHK	A*1101	9 (1)	90 (16)	2	3
AENLWVTVY	B*1801	2 (2)	78 (71)	0	1
AIFQSSMTK	A*0301, A*1101	26 (1)	75 (7)	0	1
AVDLSHFLK	A*0301, A*1101	19 (2)	75 (6)	3	1
DCKTILKAL	B*0801	22 (1)	77 (1)	0	0
DRFYKTLRA	B*1402	9 (0)	90 (6)	1	0
EIYKRWII	B*0801	22 (3)	77 (9)	0	1
ELRSLYNTV	B*0801	22 (15)	77 (60)	1	3
EVIPMFSAL	A*2601	5 (2)	94 (28)	1	1
EVKDTKEAL	B*0801	22 (15)	77 (62)	0	2
FLKEKGGL	B*0801	23 (5)	71 (10)	4	3
GEIYKRWII	B*0801	22 (3)	76 (8)	0	1
GELDRWEKI	B*4002	2 (0)	96 (2)	0	0
GGKKKYKLK	B*0801	22 (4)	76 (11)	0	1
HTQGYFPDWQ	B*5701	8 (3)	86 (25)	1	0
ILKEPVHGV	A*0201	56 (10)	45 (7)	0	0
ILKEPVHGVY	B*1501	14 (1)	87 (8)	0	0
IRLRPGGKK	B*2705	9 (2)	89 (13)	0	1
ISPRTLNAW	B*5701	7 (2)	92 (30)	1	0
IVLPEKDSW	B*5701	8 (5)	91 (35)	0	1
KAFSPEVIPMF	B*5701, B*5703	7 (0)	92 (1)	0	2
KEKGGLEGL	B*4001, B*4002	16 (5)	78 (14)	2	3
KIRLRPGGK	A*0301	11 (2)	87 (20)	0	0
KRWIILGLNK	B*2705	9 (2)	90 (20)	3	1
KYKLKHIVW	A*2402	17 (13)	81 (67)	4	4
LVGPTPVNI	A*0201	55 (0)	46 (1)	0	0
NANPDCKTI	B*5101	5 (1)	94 (16)	0	0
QASQEVKNW	B*5301, B*5701	9 (4)	90 (41)	2	5
QVPLRPMTYK	A*0301, A*1101	19 (5)	75 (20)	2	5
RLRPGGKKK	A*0301	11 (5)	87 (39)	1	2
RPNNNTRKSI	B*0702	11 (11)	69 (67)	0	1
RPQVPLRPM	B*4201	0 (0)	94 (5)	0	1
SFNCGGEFF	B*1516	0 (0)	81 (10)	0	2
SLYNTVATL	A*0201, A*0202, A*0205	60 (39)	39 (27)	3	4
TAFTIPSI	B*5101	5 (3)	95 (40)	2	0
TPGPGVRYPL	B*0702, B*4201	18 (0)	76 (3)	0	0
TPQDLNTML	B*0702, B*3910, B*4201, B*8101	21 (1)	78 (2)	0	1
TSTLQEQIGW	B*5701, B*5801	7 (6)	92 (41)	0	1
VIYQYMDDL	A*0201	56 (2)	46 (3)	0	0
VLEWRFDSRL	A*0201	55 (23)	37 (16)	2	4
VPLRPMTY	B*3501	8 (2)	86 (17)	2	3
WPTVRERM	B*0801	23 (22)	67 (53)	0	1
WRFDSRLAF	B*1503	2 (1)	90 (40)	0	7
YETEVHNVW	B*1801	2 (2)	79 (41)	0	2
YLKDQQLL	B*0801	20 (20)	61 (58)	0	1
YPGIKVRQL	B*4201	0 (0)	102 (96)	0	1
Totals		776 (246)	3631 (1173)	37	71

Table S5
