# Supplementary material for: Structured Observations Reveal Slow HIV-1 CTL Escape
Source: PLoS Genet. 2015 Feb 2;11(2):e1004914. doi: 10.1371/journal.pgen.1004914 (PMC4333731; doi:10.1371/journal.pgen.1004914)
Supplement: S6 Table — Peptides that are also in the ‘A-list’ of optimal epitopes are shown in bold. (PDF) [file pgen.1004914.s019.pdf]

Gene	Epitopes			
gag	**ACQGVGGPGHK**	AEQASQDVKNW	AEWDRVHPV	**DCKTILKAL**
	**DRFYKTLRA**	EEKAFSPEV	**EIYKRWII**	**ELRSLYNTV**
	ETINEEAAEW	**EVIPMFSAL**	**EVKDTKEAL**	FRDYVDRFYK
	**GEIYKRWII**	**GELDRWEKI**	**GGKKKYKLK**	GHQAAMQML
	GHQAAMQMLKE	GLNKIVRMY	GPGHKARVL	GQMREPRGSDI
	GVGGPGHK	HAGPIAPGQMREPRG	HPVHAGPIA	HQAISPRTL
	IEIKDTKEAL	**IRLRPGGKK**	**ISPRTLNAW**	**KAFSPEVIPMF**
	KETINEEAA	**KIRLRPGGK**	KIRLRPGGKK	**KRWIILGLNK**
	**KYKLKHIVW**	LYNTVATLY	MTNNPPIPV	**NANPDCKTI**
	NSSKVSQNY	PPIPVGDIY	QAISPRTLNAW	**QASQEVKNW**
	RDYVDRFYKTL	**RLRPGGKKK**	RLRPGGKKKY	RMYSPTSI
	RSLYNTVATLY	SEGATPQDL	**SLYNTVATL**	SPRTLNAWV
	TLNAWVKVV	TLYCVHQK	TLYCVHQRI	**TPQDLNTML**
	**TSTLQEQIGW**	VKVIEEKAF	VQNLQGQMV	WASRELERF
	YVDRFYKTL			
nef	AALDLSHFL	**AVDLSHFLK**	**FLKEKGGL**	FPVTPQVPLR
	**HTQGYFPDWQ**	**KEKGGLEGL**	LEKHGAITS	PLRPMTYK
	PLTFGWCYKL	**QVPLRPMTYK**	RPMTYKAAL	**RPQVPLRPM**
	RQDILDLWV	RRQDILDLWI	RRQDILDLWVY	RYPLTFGW
	**TPGPGVRYPL**	TPQVPLRPM	TQGYFPDWQNY	**VLEWRFDSRL**
	**VPLRPMTY**	**WPTVRERM**	**WRFDSRLAF**	YPLTFGWCF
pol	**AIFQSSMTK**	ALVEICTEM	ALVEICTEMEK	EKEGKISKI
	GIPHPAGLK	GKKAIGTVL	GPKVKQWPL	HPDIVIYQY
	IEELRQHLL	IETVPVKL	**ILKEPVHGV**	**ILKEPVHGVY**
	IRYQYNVL	**IVLPEKDSW**	KLNWASQIY	KLVDFRELNK
	KQNPDIVIY	LVGKLNWASQIY	**LVGPTPVNI**	NETPGIRYQY
	NPEIVIYQY	QIYPGIKVR	RQYDQILIEI	SPAIFQSSM
	**TAFTIPSI**	TPVNIIGRNML	TVLDVGDAY	**VIYQYMDDL**
	VPLDEDFRKY	**YPGIKVRQL**	YTAFTIPSV	
env	**AENLWVTVY**	AENLWVTVYY	DPNPQEVVL	ERYLKDQQL
	EVAQRAYR	HIGPGRAFY	IPRRIRQGL	IVNRNRQGY
	IVTRIVELL	LFCASDAKAY	LPCRIKQII	MHEDIISLW
	RGPGRAFVTI	RIKQIINMW	RIRQGLERA	**RPNNNTRKSI**
	RQGLERALL	RVKEKYQHL	RYLKDQQLL	SFEPIPIHY
	**SFNCGGEFF**	SLLNATDIAV	SVITQACPK	TAVPWNASW
	TVYYGVPVWK	VPVWKEATTT	VPVWKEATTTL	**YETEVHNVW**
	**YLKDQQLL**			

Table S6
